# Impact of a system to assist in clinical decision-making in primary healthcare in Catalonia: prescription Self Audit

**DOI:** 10.1186/s12911-022-01809-6

**Published:** 2022-03-19

**Authors:** M. Àngels Pons-Mesquida, Míriam Oms-Arias, Albert Figueras, Eduard Diogène-Fadini

**Affiliations:** 1 Unitat de Coordinació i Estratègia del Medicament (UCEM), Institut Català de la Salut, Barcelona, Spain; 2 Departament de Farmacologia, Terapèutica i Toxicologia, Universitat Autònoma de Barcelona, Barcelona, Spain; 3 Servei de Farmacologia Clínica, Hospital Universitari Vall d’Hebron, Institut Català de la Salut, Barcelona, Spain

**Keywords:** Decision support system, Primary care, Clinical safety, Electronic prescription

## Abstract

**Background:**

In 2008, in the context of a complete computerisation of medical records, the Institut Català de la Salut (ICS, Catalan Health Institute) implemented a system in its electronic clinical workstation (ECW) to assist decision-making at the prescription level. This system is known as Self Audit, and it supports physicians in reviewing the medication of their patients. Self Audit provides lists of patients presenting medication-related problems (MRPs) that have potential for improvement, and provides therapeutic recommendations that are easy to apply from the system itself. The aim of this study was to analyse the main results derived from the use of Self Audit in primary care (PC) in Catalonia, and the effect of an incentive-based safety indicator on the results obtained.

**Methods:**

A descriptive cross-sectional study was carried out to analyse variations in the MRPs detected by Self Audit during 2016, 2017, and 2018 in PC in Catalonia. The effect of a safety indicator on the results obtained was also studied. This safety indicator includes the most clinically relevant MRPs (i.e., therapeutic duplications, safety alerts from the Spanish Medicines Agency, and incidences of polymedication in patients over 65 years of age). Variation in the MRPs was measured using the differences between two evaluation points (initial and final). An MRP was considered resolved if the recommendation specified in the alert was followed. The prescriptions of 6411 PC doctors of the ICS who use the ECW and provide their services to 5.8 million Catalans through 288 PC teams were analysed.

**Results:**

Analysis of the total safety-based MRPs detected by Self Audit gave overall resolutions from April to December of 9% (21,547) in 2016, 7% (15,924) in 2017, and 1% (2392) in 2018 out of the total number of MRPs recorded in April each year. Examination of the 3 types of MRPs with the highest clinical relevance that were linked to the safety indicator gave overall resolutions of 41% in 2016 (17,358), 20% in 2017 (7655), and 21% in 2018 (8135).

**Conclusions:**

The ICS Self Audit tool assists in reducing the number of safety-based MRPs in a systematic manner, and yields superior results for the MRPs linked to a safety indicator included in the incentives of PC physicians.

## Background

In the past few decades, the development of new information and communication technologies in the field of healthcare has potentially contributed to improving the cost-effectiveness and quality of patient care. In this context, a range of technical reports from the American National Institute of Medicine have confirmed that an electronic record of healthcare activity, such as an electronic health record (EHR), together with the integration of clinical decision support systems (CDSSs) in such EHRs, constitute a guarantee of quality for the health system [1, 2]. According to several authors, CDSSs aimed at the prescription of medications have the greatest impact on improving patient safety [3]. Although a variety of different designs and functionalities exist, these systems have a common role in intelligently combining clinical knowledge and patient information, with the aims of ultimately improving the overall prescribing process. The possibility of integrating a CDSS into the EHR system has made it possible to provide medical histories with interactive signals that alert professionals to situations of risk for their patients [4], thereby helping to improve the prescription process and the overall clinical safety of patients [3, 5, 6]. Indeed, several Spanish studies have indicated that 50% of adverse events related to medication errors are avoidable [7, 8], and that the implementation of such technologies can help to reduce them.

With this background in mind, in 2008, the Institut Català de la Salut (ICS, Catalan Health Institute) integrated a combination of CDSSs into its electronic clinical workstation (ECW), namely PREFASEG, which generates online notifications when starting a treatment to prevent medication errors [9], and the Self Audit tool, which generates lists of patients presenting with active medication-related problems (MRPs). This study focuses on the Self Audit tool.

The Self Audit tool is a computerised system that is integrated into the EHR, and based on the combination of clinical with therapeutic data, it simplifies the search for patients with an MRP related to an ongoing medication, thereby facilitating changes and/or suspensions of treatment. In an agile and visual manner, it provides the professional with a list of patients with an MRP, such as a therapeutic duplication or a drug contraindicated by a previous or current pathology, thereby allowing the review and assessment of any possible change in treatment. The tool itself provides a therapeutic recommendation in each case and facilitates the management of changes and/or suspensions of treatment, without the need to leave the program. Thus, in this system, a number of aspects related to the review of a patient’s medication are systematised based on an optional and individual self-evaluation exercise. Any changes carried out are recorded in the EHR.

The MRPs are defined by a group of expert professionals from the ICS. Each year, the clinical content of the MRPs is reviewed according to the scientific information available, and, for this reason, they may vary from year to year. Each MRP is classified as high or low clinical relevance, as recommended in the literature [10].

The Self Audit tool is activated voluntarily, wherein the practitioner can use the ECW to consult the lists of patients with an MRP in his assigned population. All primary care (PC) practitioners (i.e., 100% of practitioners) use this tool at some point over the course of a year. The practitioner can also check the schedule of visits for the day, which will indicate any patients who have an MRP, and allow the doctor to take advantage of the visit to review the medication. This system therefore does not alter the workflow during the consultation [11, 12], and allows the professional to decide when is the most appropriate time to review the MRP.

From 2008 to 2016, Self Audit evolved both at the technological level and at the level of its clinical content. Initially, the tool only allowed the detection of patients with certain therapeutic duplications and/or cases of polymedication (i.e., > 10 drugs). During this period, a number of new MRP detections were incorporated into the tool, and the detection specificity was improved overall. Thus, it was not until approximately 2014 that this tool was completed in its current Self Audit configuration. In addition, the process of obtaining data to monitor the use of the tool was expensive, taking a long time to validate and debug the data until the level of quality and detail required for analysis was obtained.

Linked to the Self Audit prescription system, an incentive-based safety indicator was designed in 2008, which selected some of the most clinically relevant MRPs, and was included in the “payment for objectives” program for ICS PC physicians (N.B. according to this program, objectives are linked to annual economic incentives up to approx. 6000 €). The aim of this indicator was to promote a culture of safety in the use of medicines, and also to encourage the use of the Self Audit tool.

The aims of this study are therefore to determine the main results derived from the use of Self Audit in the Catalan PC system, and to evaluate the effect of the safety indicator on the results obtained. A further aim of this article is to provide the international audience with details regarding a computer tool aimed at improving clinical safety, which has been widely managed in the Catalan PC system by 6411 users with more than 10 years of experience. This tool is of particular importance since it helps doctors to detect patients with potential MRPs, and as a result, any ongoing treatments related to these MRPs can be reviewed and modified to benefit the health of the patient. Self Audit is a versatile and dynamic tool that can be updated with new or modified warnings as desired. Due to the considered importance of this tool to provide improvements in healthcare practice, its implementation was essentially immediate for all PC teams and professionals. Physicians refer to Self Audit as a useful tool whose usability needs, nevertheless, to be assessed.

## Methods

A descriptive, cross-sectional study was designed that began in April 2016 and continued until December 2018. This study was developed within the scope of the PC system of the ICS, which is the main provider of health services in Catalonia, a region in the northeast of Spain, and covers a population of 5.8 million inhabitants over the different Catalan territories. Overall, it serves the population through a network of 288 PC teams and 8 hospitals. The ICS is a public company that has a total of 42,374 professionals, who provide services to 80% of the population of Catalonia. Since all PC doctors of the ICS employed the Self Audit tool during routine practice, no control group was available to establish a comparison. We therefore analysed the evolution of the results over time.

### Study sample

The sample studied consisted of all the prescriptions of the 6411 ICS PC physicians (i.e., 100% of the physician staff) who used the EHRs during the study period.

### Variables and indicators

The main variable was the number of resolved MRPs. An MRP was considered “resolved” when: (1) the drug or drugs causing the MRP had been dropped from the patient's active prescription, or (2) the diagnosis was registered as resolved.

The main types of safety MRPs detected by the Self Audit tool were analysed, wherein clinically relevant MRPs that had been linked to the incentive-based safety indicator were emphasised. This safety indicator included 3 MRPs: (1) Therapeutic duplications; (2) safety alerts from the Spanish Agency for Medicines and Health Products (AEMPS, Agencia Española de Medicamentos y Productos Sanitarios), and (3) polymedication in patients over 65 years of age with some specific MRPs, wherein polymedication is defined as the case where more than 10 medicines were prescribed in 2016 and 2017, and more than 8 medications were prescribed in 2018.

The MRP related to therapeutic duplication detected patients with a non-beneficial prescription of two or more drugs that exhibit the same active principle (alone or in combination) and/or the same pharmacological action. In addition, “clinically relevant duplications” and “duplications of dose adjustments” (i.e., combinations sought with a therapeutic objective) were clearly differentiated, and only those considered relevant were linked to the safety indicator.

During the study period, the AEMPS safety alerts included the following contraindications: The “Triple Whammy”; coxibs, diclofenac, and aceclofenac; cilostazol; ivabradine; escitalopram and citalopram; trimetazidine; raloxifene and bazedoxifene; strontium ranelate; aliskiren; and canagliflozin (see Table 1).

**Table 1:** Summary of the various AEMPS safety alert criteria

Drug	Alert criteria
Citalopram	High doses:
	Above 40 mg/day
	Above 20 mg/day in patients > 65 years of age
	Above 20 mg/day in patients suffering from liver dysfunction
	Administered in combination with other drugs that also prolong the QT interval of the electrocardiogram
Escitalopram	High doses (> 10 mg/day in patients > 65 years of age)
	Administered in combination with other drugs that also prolong the QT interval of the electrocardiogram
Aliskiren	In patients with a diagnosis of diabetes mellitus II or undergoing treatment with antidiabetic drugs
	Jointly administered with ACE inhibitors
Cilostazol	In patients suffering from a health problem where its use is contraindicated, i.e., cerebral haemorrhage, severe ventricular arrhythmias, or heart failure
	Or, in concomitant treatment with:
	2 Antiplatelet agents
	Antiplatelet + oral anticoagulant
Trimetazidine	In patients with a diagnosis of extrapyramidal and movement disorders
Raloxifene or bazedoxifene	In patients suffering from any health problem where it is contraindicated, e.g., venous thromboembolism, uterine sac, endometrial cancer, or liver failure of any degree
COXIBS	In patients suffering from any health problem where it is contraindicated, e.g., ischemic heart disease, peripheral arterial disease, cerebrovascular disease, heart failure, or inflammatory bowel disease
Diclofenac or Aceclofenac	In patients suffering from any health problem where its use is contraindicated, e.g., ischemic heart disease, peripheral arterial disease, cerebrovascular disease, or heart failure
Agomelatine	In patients ≥ 75 years of age
Ivabradine	Co-administration with verapamil
“Triple Whammy” (NSAIDs + RAS inhibitors + diuretics)	In patients ≥ 75 years of age or undergoing treatment for diabetes
Canagliflozin	In patients suffering from a health problem in which it is necessary to be more careful due to an increased risk of amputation

The MRP related to incidences of polymedication detected patients older than 65 years of age with 10 or more prescribed medications (in 2016 or 2017) and with some specific MRPs, such as double antiplatelet therapy for more than 12 months, a combination of anticholinergic drugs, or other avoidable medications. In 2018, the denominator changed, and polymedication was defined as a patient with 8 or more prescribed medications.

### Data collection and analysis

The data were collected from the ECWs, where the active prescriptions of the patients are stored. The study was restricted to drugs prescribed and financed by the National Health System and employed in PC centres. The extraction of active prescription data was carried out automatically on a monthly basis, and identified the MRPs out of the prescritions of each physician detected by Self Audit.

Throughout the three years analysed (2016, 2017, and 2018), 6 points or cross-sections of information were studied. Within each year, the variations in the number of MRPs between the considered baseline data and the final data were calculated and thus the percentage variation was established. Data could not be compared between different years because the criteria that defined the detection of an MRP were different from year to year, and so such a comparison would not have been appropriate. For example, if a new pharmacological group was to be added to the “duplicate therapy” MRP in a particular year, or additional drugs were to be included in an existing duplication group, the number of MRPs related to duplicate therapies would increase.

The data obtained from the extractions carried out for the month of April were considered as the baseline data because this was the point at which the MRP detection criteria were defined and updated, and the incentive-based goals were proposed. The data obtained for the month of December were considered to be the final data since they correlated to the final month of the calendar year, and they coincided with the last evaluation point of the safety indicator. The difference between the baseline and the final data points reflected the number of resolved MRPs and the number of generated PRMs. The MRPs of individual patients were not followed over time.

The safety indicator averages the variation in the selection of the MRPs mentioned above. The effect of the incentive-based care indicator was therefore evaluated by the reduction in the number of MRPs at the PC level over a year, which was the time that the indicator remained unchanged, and which coincided with the validity of the management contract signed by the PC doctors.

To evaluate the indicator, the ability of the PC doctors to reach the goal established at the beginning of the year was measured for specific months, and was calculated from the baseline data. More specifically, the goal for each physician corresponded to a specific number of MRPs less than that existing at the beginning of the year. In the years studied, there were 2 or 3 months of the year in which the extraction of information from the active prescription MRPs was evaluated, and the ability of the doctors to reach the goal was measured. In 2016, the evaluation was carried out in September and December, while in 2017 and 2018 the evaluations were carried out in June, September, and December.

## Results

### General analysis of the MRPs of the Self Audit tool

The data extractions corresponding to the months of December 2016, December 2017, and December 2018 showed that the ECW had registered 9.5, 9.6, and 9.7 million active prescriptions, respectively. In these months, 210,916 MRPs (December 2016), 227,856 MRPs (December 2017), and 230,959 MRPs (December 2018) were identified. Based on these data, it was observed that the percentage of MRPs studied with respect to the total number of active prescriptions represented 2.2% in 2016 and 2.4% in 2017 and 2018.

Upon analysis of the total clinical safety MRPs detected by Self Audit, an overall resolution of 9% (21,547) was observed in 2016, while resolutions of 7% (15,924) and 1% (2392) were found in 2017 and 2018, respectively (see Table 2).

**Table 2:** Problems related to medications detected by Self Audit: April 2016–December 2018

Problem detected by Self Audit	Year 2016*			
	Apr 2016	Dec 2016	Variation	Percentage (%)
Duplicate therapies	46,242	41,589	− 4653	− 10
AEMPS safety alerts	13,521	6849	− 6672	− 49
Contraindications due to medical devices and/or clinical variables	37,359	37,421	62	0
Treatment duration				
Bisphosphonates ≥ 5 years	8246	7434	− 812	− 10%
Double anti-aggregation ≥ 12 months	4805	4332	− 473	− 10%
Drugs advised against in geriatrics	88,393	83,638	− 4755	− 5
Combination of anticholinergic drugs	2913	2320	− 593	− 20
Avoidable medication	30,984	27,333	− 3651	− 12
Total number of problems detected	232,463	210,916	− 21,547	− 9

Problem detected by Self Audit	Year 2017			
	Apr 2017	Dec 2017	Variation	Percentage (%)
Duplicate therapies	65,679	59,536	− 6143	− 9
AEMPS safety alerts	11,212	9935	− 1277	− 11
Contraindications due to medical devices and/or clinical variables	44,687	44,000	− 687	− 2
Treatment duration				
Bisphosphonates ≥ 5 years	6386	4964	− 1422	− 22%
Double anti-aggregation ≥ 12 months	4394	4508	114	3%
Drugs advised against in geriatrics	82,252	79,187	− 3065	− 4
Combination of anticholinergic drugs	2184	1819	− 365	− 17
Avoidable medication	26,986	23,907	− 3079	− 11
Total number of problems detected	243,780	227,856	− 15,924	− 7

Problem detected by Self Audit	Year 2018			
	Apr 2018	Dec 2018	Variation	Percentage (%)
Duplicate therapies	65,377	64,650	− 727	− 1
AEMPS safety alerts	7046	5441	− 1605	− 23
Contraindications due to medical devices and/or clinical variables	45,175	46,469	1294	3
Treatment duration				
Bisphosphonates ≥ 5 years	4552	4123	− 429	− 9%
Double anti-aggregation ≥ 12 months	4631	4630	− 1	0%
Drugs advised against in geriatrics	79,225	79,384	159	0
Combination of anticholinergic drugs	1854	1736	− 118	− 6
Avoidable medication	25,491	24,526	− 965	− 4
Total number of problems detected	233,351	230,959	− 2392	− 1

*In the three years studied, April was taken as the baseline data because it is the time at which the definitions of the MRPs were updated according to the consensus of a group of experts, and it also is the month in which the incentive-based goals were proposed

Upon the analysis of all clinical safety MRPs detected by Self Audit, overall resolutions of 9% (21,547), 7% (15,924), and 1% (2392) were observed in 2016, 2017, and 2018, respectively. Subsequent analysis of the resolutions of the different MRPs throughout the whole study period (i.e., 2016–2018) showed an overall trend towards resolution, especially in the cases where AEMPS safety alerts were implemented, since this resulted in 49% resolution of the cases in 2016, 11% in 2017, and 23% in 2018.

The behaviours of the specific MRPs were then examined in further detail. More specifically, in April 2016, a total of 46,242 duplications were detected, while in December of the same year, such duplications had been reduced by 10% (i.e., to a total of 41,589). However, there was a significant increase in the absolute number of duplications detected in 2018 (65,377 in April and 64,650 in December), with a reduction of only 1% being achieved throughout the year. In addition, it was found that the MRPs related to drugs not recommended for use in geriatric patients exhibited reductions of 5% (4755 cases) in 2016 and 4% in 2017 (3065 cases), although no reduction was found in 2018. Furthermore, the MRP related to avoidable medications (including chondroprotectors and citicoline) showed a reduction of 3651 cases in 2016 (− 12%), 3079 cases in 2017 (− 11%), and 965 cases in 2018 (− 4%).

Taking the last analysis point, namely that of December 2018, 34% of the 230,959 MRPs detected were due to the use of drugs not recommended for use in geriatric patients, while 28% were attributed to therapeutic duplications, and 20% were due to pathological contraindications. As a result, these three MRPs accounted for more than three-quarters of the overall MRPs detected at this point (i.e., 190,503 cases, 82.5%).

### Analysis of clinically relevant MRPs linked to the PA safety indicator

**Fig. 1:**
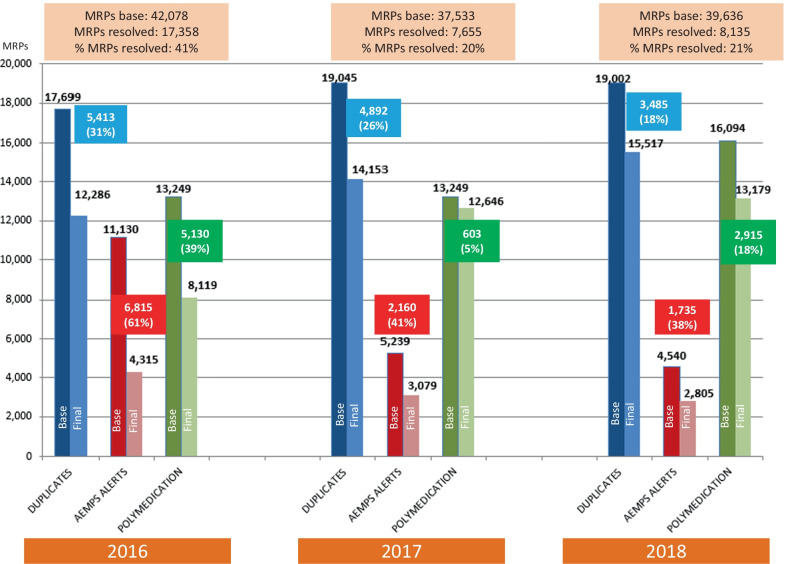
Variation in the MRPs linked to the 2016–2018 incentive-based safety indicator

In the period studied, 41,492 MRP cases were resolved in the Self Audit tool, of which 80% (33,148) were linked to the safety indicator. Upon examination of the 3 types of MRPs linked to this indicator, joint resolutions of 41% in 2016 (17,358), 20% in 2017 (7655), and 21% in 2018 (8135) were observed, as detailed detail in Fig. 1. However, despite these promising percentages of resolution, the total number of these three MRPs increased from 24,720 in December 2016 to 31,501 in December 2018.

With reference to the specific MRPs, in the case of therapeutic duplications, the resolution of 5413 cases in 2016, 4892 cases in 2017, and 3485 cases in 2018 was achieved. In addition, when considering the MRPs related to the AEMPS alert, 6815 cases were resolved in 2016, which dropped to 2160 cases in 2017, and 1735 cases in 2018. Furthermore, for the MRP related to polymedication, the corresponding reductions were 5130 cases in 2016, 603 cases in 2017, and 2915 cases in 2018.

**Fig. 2:**
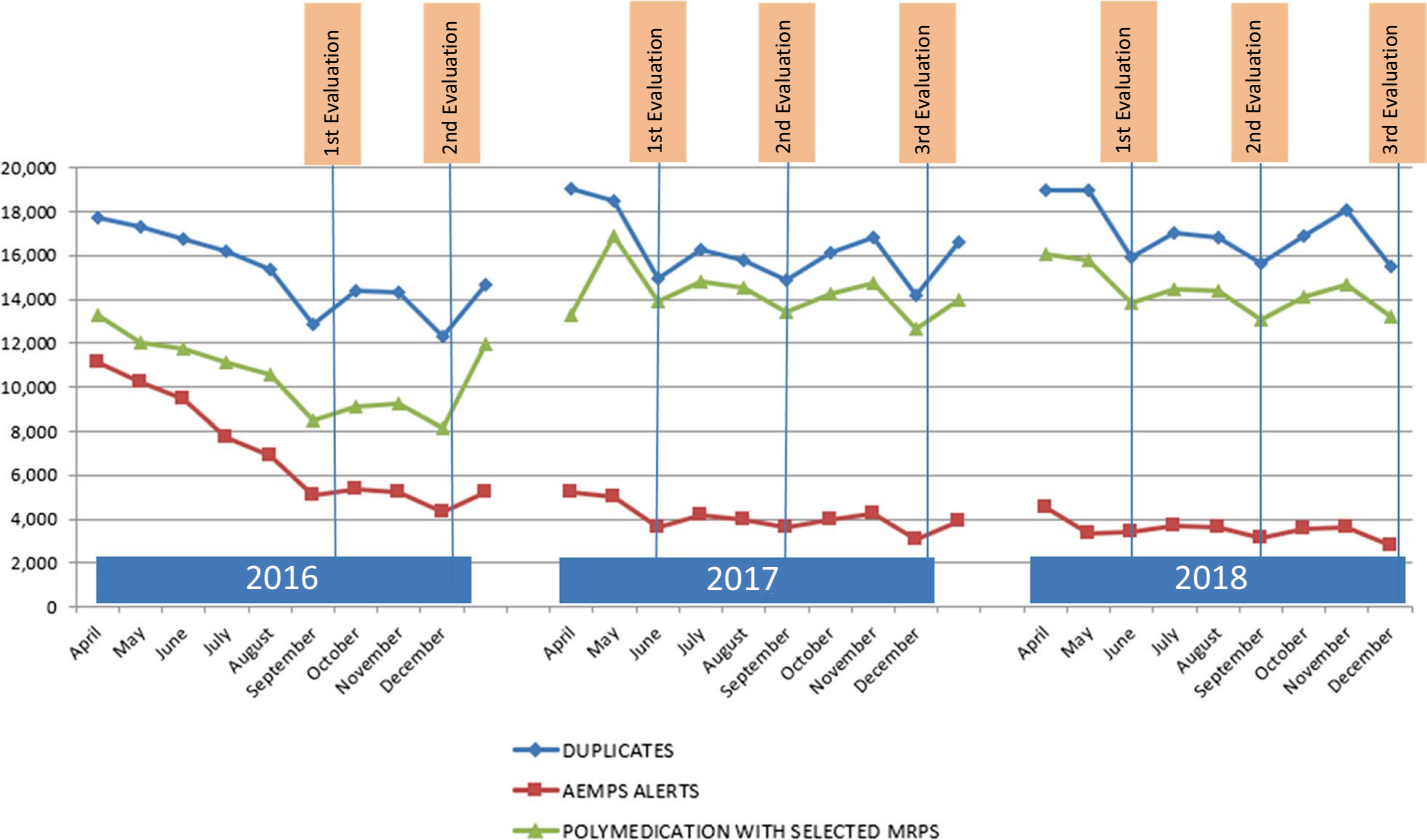
Annual performance of the 2016–2018 incentive-based safety indicator

The annual data for the safety indicator showed that at the time of the evaluation, there was a greater decrease in the number of MRPs, while after each evaluation point there was a rebound in the number of cases, as can be seen in Fig. 2. The most pronounced rebound was observed after the December evaluation point, as will be discussed later.

Upon analysis of the detail relating to the AEMPS safety alerts (Table 3), it was observed that the Triple Whammy represented 89% (9767/11,035) of the total alerts included in 2016, 80% (4123/5153) of those in 2017, and 73% (3260/4460) of those in 2018. Its reduction percentage ranged from 66% in 2016 to 43% in 2018.

**Table 3:** Evolution and reduction percentages by type of AEMPS safety alert

Year	2016				2017				2018			
AEMPS ALERT	Apr	Dec	Variation	Percentage (%)	Apr	Dec	Variation	Percentage (%)	Apr	Dec	Variation	Percentage (%)
Triple whammy	9767	3356	− 6411	− 66	4123	2242	− 1881	− 46	3260	1850	− 1410	− 76
Coxibs	407	282	− 125	− 31	349	240	− 109	− 31	384	283	− 101	− 36
Diclofenac	325	144	− 181	− 56	189	109	− 80	− 42	189	96	− 93	− 97
Cilostazol	115	105	− 10	− 9	107	82	− 25	− 23	101	78	− 23	− 29
Ivabradine	102	88	− 14	− 14	87	73	− 14	− 16	82	65	− 17	− 26
Aceclofenac	82	60	− 22	− 27	69	43	− 26	− 38	51	28	− 23	− 82
Agomelatine	74	62	− 12	− 16	78	69	− 9	− 12	81	63	− 18	− 29
Escitalopram	55	51	− 4	− 7	54	61	7	13	60	45	− 15	− 33
Citalopram	41	34	− 7	− 17	48	47	− 1	− 2	59	46	− 13	− 28
Trimetazidine	40	27	− 13	− 33	34	24	− 10	− 29	34	26	− 8	− 31
Raloxifene and Bazedoxifene	14	9	− 5	− 36	10	7	− 3	− 30	9	8	− 1	− 13
Strontium ranelate	8	9	1	13	3	0	− 3	− 100	NA	NA	NA	NA
Aliskiren	5	4	− 1	− 20	2	3	1	50	3	2	− 1	− 50
Canagliflozin	NA	NA	NA	NA	NA	NA	NA	NA	147	126	− 21	− 17
TOTAL ALERTS	11	4	− 7	− 62	5	3	− 2	− 42	4460	2716	− 1744	− 64

NA: Not applicable

As indicated in Table 3, the reduction in the number of cases related to the AEMPS safety alert for diclofenac was 56% in 2016, while the reduction for acecloflenac was 27%. In 2017, the corresponding reductions for diclofenac and acecloflenac were 42 and 38%, respectively, and in 2018, they were 49 and 45%. Furthermore, the reductions in cases related to alerts for the coxibs were 31, 31, and 26% in 2016, 2017, and 2018, respectively, while the corresponding reductions for the cilostazol alert were 9% in 2016 but 23% in 2017 and 2018. The remainder of alerts represented few cases in absolute numbers.

Following analysis of the therapeutic duplications (Table 4), it was found that 70% fell into 10 pharmacological groups out of a total of 63. Of these 10 groups, the renin-angiotensin system inhibitors stood out particularly (2828 detected in December 2016, and 1939 detected in December 2018), along with non-steroidal anti-inflammatory drugs (2084 and 1969), long-acting benzodiazepines (1796 and 1854), and inhaled glucocorticoids (1741 and 1771). These four groups of drugs represented 68% of the duplicities detected in December 2016, and 48.5% of those detected in December 2018. The groups that experienced an increase in detected duplications over the same period were the antidepressants (1417 and 1701 in December 2016 and December 2018, respectively), the urinary antispasmodics (1219 and 2020), and the thiazide diuretics (943 and 1595). Thus, these three groups accounted for 29.1% of the duplications detected in December 2016, and 34% of those detected in December 2018.

**Table 4:** Top 10 prescribed duplicate groups in 2017 and 2018, including June–December variations

Duplicate group*	June 2017	Dec 2017	Variation	Percentage (%)	June 2018	Dec 2018	Variation	Percentage (%)
Renin-angiotensin system inhibitors	2458	2141	–317	–13	2107	1939	–168	–9
Anti-inflammatories	2154	1880	–274	–13	2203	1969	–234	–12
Long-acting benzodiazepines	2032	1911	–121	–6	2064	1854	–210	–11
Inhaled glucocorticoids	1551	1616	65	4	1583	1771	188	11
Alpha adrenergic antagonists	1273	1299	26	2	1337	1346	9	1
Gastric protectors	1425	1162	–263	–18	1364	1276	–88	–7
Other anti-depressants I	1467	1389	–78	–5	1562	1702	140	8
Urinary antispasmodic agents	1655	1547	–108	–7	1876	2020	144	7
Thiazide diuretics	964	823	–141	–15	2085	1595	–490	–31
Paracetamol (analgesic)	545	668	123	23	523	570	47	8

For technical reasons, the April data were not recorded at the level of detail required for the duplication group and so they have not been included in the table. The data were analysed in June and December, at which points they met the level of quality and detail required for analysis

*For each duplicate group, all available active prescription data are shown, which coincide with the evaluation points

Through analysis of the 20 groups of therapeutic duplications with the highest number of cases, it was observed that in 2017, the groups that presented the greatest degrees of reduction in cases were the gastric protectors (− 18%), the thiazide diuretics (− 15%), and the metamizole-type analgesics (− 14%, data not shown). In 2018, the groups exhibiting the greatest degrees of reduction were the sulfonamide diuretics (− 75%), the thiazide diuretics (− 24%), and the beta-blockers (− 12%).

## Discussion

The prescription Self Audit system is a clinical management computer tool, aimed at increasing the quality of care by giving support to health professionals in the move towards the safe and effective prescription of drugs.

The main finding of this study was that Self Audit is positioned as a CDSS, which is widely used among the doctors of the Catalan PC system to help identify and resolve safety PRMs in a systematic manner, and leads to superior results for the MRPs linked to the incentive-based safety indicator developed for PC physicians. Therefore, we think that results could be improved by implementing awareness strategies and providing feedback to physicians. This should could be followed by more specific recommendations, which should be repeated and regularly inspected These hypothesis, of course, need to be verified.

In the period studied, 41,492 cases of potential safety problems that could affect patient health were resolved, of which 80% (33,148) were linked to the safety indicator. In general terms, the percentage of MRPs detected by Self Audit ranged between 2.2 and 2.4% of the active prescriptions in the ECW (> 9 million) during the years studied. It should be noted there that the detection of an MRP depended on the defined clinical content, and in the case of Self Audit, this content was updated annually. As a result, data could not be compared between different years.

Although the number of MRPs resolved was significant, the percentage of MRPs resolved each year with respect to the number detected by Self Audit was low, namely less than 10%. In addition, the numbers of some MRPs increased over time, as in the case of therapeutic duplications (> 60,000 cases pending resolution in 2018) and contraindications due to pathologies (> 46,000 cases pending resolution in 2018). This result indicates that significant numbers of MRPs must still be solved, and so supports the need to design interventions that contribute to improving the prescribing attitude. Moreover, it will be necessary to analyse the reasons for these increases and/or low resolution levels, in addition to assessing the requirement to make the detections of some MRPs more specific, and/or to more clearly detail the therapeutic recommendations that are offered.

As pointed out in a previous study [9], Self Audit is common among PC physicians. However, during the study period examined herein, resolution of the different Self Audit MRPs was found to be heterogeneous and irregular. More specifically, some MRPs presented high percentages of resolution, such as those related to the AEMPS alerts. This was perhaps due to the fact that there is greater response from professionals when a safety alert is issued by a regulatory body [13]. Although the use of AEMPS alerts resulted in a significant reduction in the number of cases in 2018, it should also be pointed out that part of this reduction was due to a change in the clinical content, wherein the warnings for citalopram and escitalopram became more specific (i.e., alerts were only issued if these drugs were prescribed together with other medications that prolong the QT interval), and so these medications generated fewer detections.

Several Self Audit MRPs also showed increases in the number of MRP in the period of study, and this could be attributed to various factors, that need to be validated. For example, the increase in therapeutic duplications over the years could be explained by the fact that new groups of drug duplications or new active ingredients marketed in different groups had been included.

Another MRP that attracted attention due to its negligible decrease, or even a certain increase, was the contraindication group. The results related to this MRP can be explained by considering that the content of this MRP changed substantially during the study period. More specifically, in 2017, the contents were expanded to include contraindications due to the altered values of some clinical variables (e.g., potassium and glomerular filtration), while in 2018, a global update of the contraindications took place, thereby resulting in increased detections of this MRP. However, the reasons behind their low resolution percentages require further investigation. It is possible that this could be attributed to a lack of specificity of the warning and/or recommendation, since different authors [14, 15] have supported the fact that giving a clear and precise recommendation constitutes one of the success criteria of the CDSS. It is also a possibility that the recommendations provided in some cases suggest that a clinical follow-up should be carried out, and therefore do not result in the withdrawal of any medication. Under such circumstances it would be assumed that the MRP is not resolved, despite the fact that the recommendation is actually being followed.

In contrast, the MRP related to the drugs not recommended for use in geriatric patients exhibited a particularly low or no reduction during the years of study. In this case, there was no change in the clinical content; however, the low resolution percentage was attributed to this being an MRP of low clinical relevance, and the fact that the literature [16–18] does not consider that the use of these drugs are fully contraindicated in older patients, but instead it is simply recommended that they not be used. The same argument would serve to justify the low resolution of the MRP related to avoidable medications (i.e., chondroprotectors and citicoline). Thus, when doctors are faced with different MRPs, they prioritise those that are clinically more relevant, or that can be solved more rapidly or with less effort [19]. Another explanation to consider for the low resolution percentages associated with these two MRPs is that they are not included in the safety indicator.

It is also known that healthcare practice generates multiple incidences of medication, which suggests that the total resolution of MRPs through the Self Audit tool was considerably higher than that indicated in the results of the study. This could be attributed to the resolution of some MRPs at the same time as new ones being created; this behavior is not reflected in the current study. It should also be noted that the patients with MRPs were not followed over time, but instead, the existing MRPs under active prescription were compared at two different times within a year. In addition, it must be taken into account that the world population is continually aging, and this is accompanied by a greater incidence of pathologies, and an increase in the use of medications [20–22]. Indeed, it has recently been reported that if recent health trends continue, Spain is on its way to becoming the leading country in terms of the highest life expectancy in 2040 (i.e., 85.8 years) [23]. As a result of such aging, the greater incidence of multiple associated pathologies results in an increased consumption of drugs, which favours the appearance of increasingly complex therapeutic regimens. This in turn is associated with a higher frequency of adverse effects, interactions, and hospital admissions, in addition to a poorer quality of life and a lack of treatment compliance [24].

In terms of the health impact, a reduction in the number of MRPs can be translated into the avoidance of adverse drug effects in patients, which are known to have a considerable impact on patient morbidity and mortality [3], in addition to increasing the average cost of care [5], increasing the number of visits to primary healthcare centres, and increasing hospital admissions [25].

Upon analysis of the MRPs linked to the safety indicator, it was observed that the resolution of these MRPs was significantly higher than that of the general Self Audit data. This could be explained by considering that the included MRPs are of greater clinical relevance, or that it is an economically incentivised indicator. Another key point is that the reduction in cases decreased year on year, both in terms of the absolute number and the percentage. One explanation for this could be that the composition of the indicator varied each year, and therefore the target population for intervention was different, and could have been smaller. Another hypothesis that was considered was that the baseline starting point improved over time, until it reached a point where further improvements were difficult to achieve.

The annual plots obtained for the evolution of the MRPs linked to the indicator clearly showed a decrease in cases at the time of evaluation. The highest degree of MRP resolution occurred at the end of December, and this was accounted for by considering that historically, this indicator had always been evaluated in a single evaluation point at the end of the year. Every January, a relevant increase in cases was observed, although the baseline point was not reached, and so it was assumed that the professionals were indeed acquiring a certain culture of safety, and that the MRPs generated during the daily healthcare practice were being solved. The results of our study therefore appear to be in line with a previous study, wherein the authors suggest that incentive-based systems could influence physicians, and ultimately lead to an improvement in healthcare provision [26, 27]. However, in a Cochrane review by Scott et al. [28] regarding this point, it was concluded that there was insufficient evidence to indicate whether financial incentives had a positive impact on the quality of care in PC systems.

On the other hand, it is known that intervention strategies based on improving the prescription of drugs through audits and feedback to physicians have improved the quality of care, wherein such feedback includes information corresponding to their own patients, in addition to specific improvement recommendations; these strategies are repeated and supervised by other colleagues [11, 12, 29, 30]. In this context, the ICS can highlight that this individualised feedback is standard practice for its pharmacists and PC pharmacologists [31, 32]. However, the collected data show that there is significant room for improvement, as the number of MRPs that are pending resolution is considerable. It is therefore evident that it will be necessary to design specific intervention strategies to attain a change in the prescribing attitude. Such strategies could include the close monitoring of data at an individual level, training support, and continuous review of the clinical contents to ensure that they are specific and that they are accompanied by concrete therapeutic recommendations [33].

Furthermore, it does not go unnoticed that it is necessary to evolve and improve the Self Audit tool at a technological level to make it more user-friendly and intuitive, and to impart a greater degree of integration with the patient's medical records. Moreover, this tool should be provided with artificial intelligence elements that possess more agile algorithms for information interpretation, and to facilitate decision making.

Finally, it should be noted that one of the main limitations of this study is that there is no follow-up over time for patients with certain MRPs, thereby preventing us from knowing how many MRPs persist over time, how many are new MRPs, and how many MRPs return or reappear after a while. Indeed, such follow-ups would be beneficial to allow the consequences on the patient's health to be evaluated. Likewise, continuous changes in the clinical contents also made it difficult to analyse the temporal evolution of each type of MRP.

## Conclusions

The Self Audit clinical decision support system developed by the Institut Català de la Salut helps to systematically identify and resolve safety medication-related problems (MRPs) in a systematic manner, wherein superior results were obtained for the MRPs linked to a safety indicator that is included in the incentives of primary care physicians. However, it is noted that significant room for improvement exists in the prescribing attitude, and as a result, additional medical awareness strategies will be necessary, as well as improvements to the tool itself. Such improvements should be based at a technical level and should be aimed at increasing specificity in MRP detection and subsequent recommendations. Finally, we believe that in the context of clinical safety, the implementation HER tools similar to Self Audit could be a useful and beneficial healthcare strategy that could benefit patients from other healthcare systems worldwide.

## Data Availability

The datasets used and/or analysed during the current study are available from the corresponding author on reasonable request.

## Abbreviations

AEMPS: Spanish Agency for Medicines and Health Products (Agencia Española del Medicamentos y Productos Sanitarios)
CDSS: Clinical decision support system
ECW: Electronic clinical workstation
EHR: Electronic health record
ICS: Institut Català de la Salut (Catalan Health Institute)
MRP: Medication-related problem
PC: Primary care
